# A New Quinone-Based Inhibitor of Mitochondrial Complex I in D-Conformation, Producing Invasion Reduction and Sensitization to Venetoclax in Breast Cancer Cells

**DOI:** 10.3390/antiox12081597

**Published:** 2023-08-10

**Authors:** Matías Monroy-Cárdenas, Víctor Andrades, Cristopher Almarza, María Jesús Vera, Jorge Martínez, Rodrigo Pulgar, John Amalraj, Ramiro Araya-Maturana, Félix A. Urra

**Affiliations:** 1 Interdisciplinary Group on Mitochondrial Targeting and Bioenergetics (MIBI), Talca 3480094, Chile; 2 Instituto de Química de Recursos Naturales, Universidad de Talca, Casilla 747, Talca 3480094, Chile; 3 Laboratorio de Plasticidad Metabólica y Bioenergética, Programa de Farmacología Molecular y Clínica, Instituto de Ciencias Biomédicas (ICBM), Facultad de Medicina, Universidad de Chile, Independencia 1027, Casilla 7, Santiago 7810000, Chile; 4 Network for Snake Venom Research and Drug Discovery, Santiago 7810000, Chile; 5 Laboratorio de Biología Celular, Instituto de Nutrición y Tecnología de los Alimento (INTA), Universidad de Chile, Santiago 7830490, Chile; 6 Laboratorio de Genómica y Genética de Interacciones Biológicas (LG2IB), Instituto de Nutrición y Tecnología de los Alimento (INTA), Universidad de Chile, El Líbano 5524, Santiago 7830490, Chile

**Keywords:** anti-cancer agents, quinones, oxidative phosphorylation, Complex I, electron transport chain, Rho-0 cells

## Abstract

Mitochondrial Complex I plays a crucial role in the proliferation, chemoresistance, and metastasis of breast cancer (BC) cells. This highlights it as an attractive target for anti-cancer drugs. Using submitochondrial particles, we identified FRV–1, an *ortho*-carbonyl quinone, which inhibits NADH:duroquinone activity in D-active conformation and reduces the 3ADP state respiration dependent on Complex I, causing mitochondrial depolarization, ATP drop, increased superoxide levels, and metabolic remodeling towards glycolysis in BC cells. Introducing methyl groups at FRV–1 structure produced analogs that acted as electron acceptors at the Complex I level or increased the inhibitory effect of FCCP-stimulated oxygen consumption rate, which correlated with their redox potential, but increased toxicity on RMF-621 human breast fibroblasts was observed. FRV–1 was inactive in the naphthoquinone oxidoreductase 1 (NOQ1)-positive BC cell line, MCF7, but the sensitivity was recovered by dicoumarol, a NOQ1 inhibitor, suggesting that FRV–1 is a NOQ1 substrate. Importantly, FRV–1 selectively inhibited the proliferation, migration, and invasion of NQO1 negative BC cell, MDA-MB-231, in an OXPHOS- and ROS-dependent manner and sensitized it to the BH3 mimetic drug venetoclax. Overall, FRV–1 is a novel Complex I inhibitor in D-active conformation, blocking possibly the re-activation to A-state, producing selective anti-cancer effects in NQO1-negative BC cell lines.

## 1. Introduction

Breast cancer (BC) comprises several biologically distinct subtypes that have variations in the presence of estrogen receptors (ER), progesterone receptors (PR), and human epidermal growth factor receptor-2 (HER2). In triple-negative breast cancer (TNBC), which lacks ER, PR, and HER2, patients develop pulmonary, hepatic, and cerebral metastases more frequently than other breast cancer sub-types. Current chemotherapy treatments do not brake the metastasis, highlighting the need to obtain compounds that inhibit migratory and invasive abilities, which have been named migrastatics [1].

Mitochondria of breast cancer cells are essential for supporting the metastatic cascade by upregulation of electron transport chain (ETC) genes, increased oxidative phosphorylation (OXPHOS), and superoxide production [2,3,4]. Notably, TNBC cells have reduced Complex I-dependent respiration and a diminished spare respiratory capacity compared to triple-positive BC cells [5]; however, it maintains the NAD/NADH ratio for metastasis [6]. This suggests a high vulnerability towards inhibitors of mitochondrial respiration, reducing proliferation, tumor growth, and metastasis [7,8].

NADH-ubiquinone oxidoreductase, also named Complex I, is the largest unit of the mitochondrial electron transport chain and contributes about 40% of the proton motive force necessary for mitochondrial ATP synthesis [9]. This respiratory complex can adopt two catalytically and structurally different states, known as active (A) and de-active, dormant (D) conformation. Although these conformations were considered an experimental artifact, recent evidence suggests that the catalytically active A-state can transit to a D-state in several physiological contexts, such as a reduced oxygen availability or NO production, reducing in this way the superoxide production and electron transfer for respiration [10,11]. The conformational rearrangements of the A/D transition involve the mitochondrially encoded subunits ND1 and ND3, besides the accessory subunit NDUFA9, all located in the quinone-binding site. In D-state, a loss of communication between proton pumping (P-module) and redox modules occurs, blocking the terminal electron transfer from the N2 FeS cluster to ubiquinone [12]. It has been identified that the Cys39 of the ND3 subunit is a critical residue that is exposed toward the matrix and is only accessible for chemical modification in the D-form [10], which prevents the re-activation of Complex I [13].

Notably, some small molecules and FDA-approved drugs can differentially interact with Complex I in an A/D-state conformation manner. Rotenone inhibits the Complex I activity by binding to A-state, lacking effects on D-state [14,15], and metformin binds to Complex I in D-state possibly by reaction with Cys39 [14,16]. However, the structural requirements of compounds that act on the D-active state and the effect on mitochondrial bioenergetics of TNBC cells are largely unknown.

Previously, we have described that an *ortho*-carbonyl hydroquinone motif can produce inhibitors of Complex I-dependent respiration and OXPHOS uncouplers with antiproliferative and migrastatic effects in BC cells [17,18,19]. In line with this, the chemical reactivity of FRV–1, an *ortho*-carbonyl bicyclic quinone, has been studied for several years [20,21,22,23]. The quinone moiety is converted into a strong electrophile and oxidant agent when conjugated with a carbonyl group [24]; however, their pharmacological activities and interactions with biological targets are unknown. In this work, we identified FRV–1 as a novel Complex I inhibitor that acts on D-state, exhibiting anti-cancer effects in BC cells.

## 2. Materials and Methods

### 2.1. Compounds and Reagents

All chemical reagents were bought from commercial suppliers, reagent grade, and were used without further purification. All reagents used in the pharmacological section were obtained from Sigma-Aldrich Corp. (St. Louis, MO, USA). Stock solutions of all quinones were prepared in dimethyl sulfoxide (DMSO).

### 2.2. Synthetic Procedures

Duroquinol was synthesized by reduction from duroquinone as previously described [17]. ^1^H and ^13^C NMR spectra were obtained on a Bruker Avance 400 NMR spectrometer operating at either 400.13 MHz (^1^H) or 100.61 MHz (^13^C). Chemical shifts are reported as ppm downfield from TMS for ^1^H NMR and relative to the central CDCl_3_ resonance (77.0 ppm) for ^13^C NMR. All melting points were uncorrected and were determined on an Electrothermal 9100 apparatus. High-resolution mass spectra were obtained on a Bruker compact QTOF-MS spectrometer. Silica gel 60 (70–230 mesh) was used for flash preparative column chromatography, and TLC aluminum foil 60F254 for analytical TLC. 

Hydroquinones FRHV–1-FRHV–6 and quinones FRV–1-FRV–6 were synthesized by published procedures [17,18,19,20,21,22,23,24]. Spectra of the reported compounds are shown in Figure S1.

#### General Procedure for the Synthesis of Quinones FRV–1-FRV–6

The 1-(2,5-dihydroxy-3,4-dimethylphenyl)ethanone, 1-(2,5-dihydroxyphenyl)propan-1-one, and 1-(2,5-dihydroxy-3,4-dimethylphenyl)propan-1-one were obtained using the already reported methodology [25,26] although changing microwave heating by a conventionally heated reaction, in a sealed-vessel reactor (Monowave 50, Anton Paar, Graz, Austria) as follows: To a 10 mL process vial, were added one equivalent of hydroquinone or dimethylhydroquinone, 1.5 equivalent of acetic acid or propanoic acid, 4 mL of boron trifluoride dihydrate, and a magnetic stir bar. The reaction mixture was heated at 140 °C for 30 min, and then the mixture was poured into a saturated NaHCO_3_ solution. Then, it was extracted with ethyl acetate, and the organic phase was dried with anhydrous sodium sulfate, later filtered, and the solvent evaporated under a vacuum. Afterward, acyl hydroquinones were purified by flash chromatography with 8:1 hexane:ethyl acetate as the mobile phase. These compounds, besides commercial acetylhydroquinone (1), were used as starting products for the synthesis of the bicyclic quinones as follows. The corresponding acylhydroquinone was oxidized with Ag_2_O in dichloromethane, and the filtered solution was drip added on a solution of the corresponding enamine to afford the cyclic O,N-acetal, which were not isolated; the crude products were refluxed in acidic ethanol for two hours, afterward, the reaction mixture was poured over a mixture of ice water, the suspension was extracted with ethyl acetate (3 × 30 mL), obtaining the corresponding bicyclic hydroquinones FRHV–1–FRHV–6, which were purified by column chromatography using 4:1 hexane:ethyl acetate as eluent.

The quinones FRV–1-FRV–6 were obtained by oxidation of the corresponding bicyclic hydroquinones. A suspension, in CH_2_Cl_2,_ of corresponding hydroquinone and Ag_2_O was stirred for one hour at room temperature and then filtered through celite. The solvent was evaporated under vacuum, obtaining the corresponding quinones, which were purified by flash column chromatography using 4:1 hexane:ethyl acetate as mobile phase.

**Figure 1:**
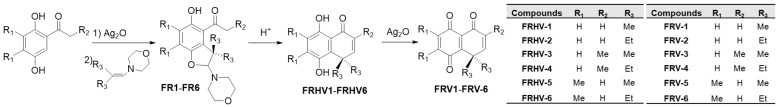
Synthesis of compounds.

In this way, the already reported compounds 5,8-dihydroxy-4,4-dimethylnaphthalen-1(4*H*)-one (FRHV–1), 5,8-dihydroxy-4,4,-diethylnaphthalene-1(4*H*)-one (FRHV–2), 5,8-dihydroxy-2,4,4-trimethylnaphthalen-1(4*H*)-one (FRHV–3), 5,8-dihydroxy-4,4,6,7-tetramethylnaphthalen-1-one(4*H*)-(FRHV–5), and the new analogs were obtained. Figure 1 shows the synthetic routes and chemical structures for compounds FRHV and FRV, respectively.

The *4,4-diethyl-5,8-dihydroxy-2-methylnaphthalen-1(4H)-one (FRHV–4)*, yellow solid (23% yield). ^1^H NMR δ: 0.45 (t, *J* = 7.4 Hz, 6H, 2xCH_3_), 1.49 (dq, *J*_1_ = 7.4, *J*_2_ = 14.9 Hz, 2H, 2xCHH), 1.96 (d, *J* = 0.9 Hz, 3H, CH_3_), 2.67 (dq, *J*_1_ = 7.5 Hz *J*_2_ = 14.9 Hz, 2H, 2xCHH), 4.50 (s, OH), 6.39 (d, *J* = 0.9 Hz, 1H, 3-H), 6.70 (d, *J* = 8.8 Hz, 1H), 6.77 (d, *J* = 8.8 Hz, 1H), 12.91 (s, OH), ^13^C RMN δ: 9.43, 15.59, 30.70, 48.03, 115.78, 117.88, 123.41, 131.87, 134.47, 145.07, 155.90, 156.90, 192.35), M.p. 176.2–178.4 °C. HRMS (ESI) *m*/*z* calcd. For C_15_H_18_O_3_ [M+H]^+^: 247.1329, found: 247.1333.

The *4,4-diethyl-5,8-dihydroxy-6,7-dimethylnaphthalen-1(4H)-one (FRHV–6).* ^1^H NMR δ: 0.54 (t, *J* = 7.5 Hz, 6H), 1.57 (dq, *J*_1_ = 7.5 Hz, *J_2_* = 14.0 Hz, 2H), 2.24 (s, 3H), 2.25 (s, 3H), 2.77 (dq, *J*_1_ = 7.5 Hz, *J*_2_ = 14.0 Hz, 2H), 4.40 (s, OH), 6.46 (d, *J* = 10.1 Hz, 1H), 6.61 (d, *J* = 10.1 Hz, 1H), 13.25 (s, OH). ^13^C NMR δ: 9.44, 11.60, 13.06, 30.77, 48.57, 115.32, 123.52, 127.95, 128.53, 131.17, 143.30, 155.32, 159.21, 191.83. M.p. 176.2–178.4 °C. HRMS (ESI) *m*/*z*: calcd. for C_16_H_20_O_2_ [M+H]^+^: 261.1485, found: 261.1489.

The 8,8-dimethylnaphthalene-1,4,5(8H)-trione (FRV–1), 8,8-diethylnaphthalene-1,4,5(8H)-trione (FRV–2), and 6,8,8-trimethylnaphthalene-1,4,5(8H)-trione (FRV–3), have been already reported [27,28,29], the new analogs were obtained as follows:

The *8,8-diethyl-6-methylnaphthalene-1,4,5(8H)-trione (FRV–4).* Reddish liquid (86% yield). ^1^H-NMR δ: 0.60 (t, *J* = 7.57 Hz, 6H, 2xCH_3_), 1.69 (dq, *J*_1_ = 7.5 Hz, *J*_2_ = 15.0 Hz, 2H, 2xCHH), 1.99 (s, 3H, CH_3_), 2.50 (dq, *J*_1_ = 7.4 Hz, *J*_2_ = 15.0 Hz, 2H, 2xCHH), 6.38 (s, 1H, 6-H), 6.74(d, *J* = 10.5 Hz), 6.77(d, *J* = 10.5 Hz). ^13^C NMR δ: 9.44, 15.72, 32.64, 48.33, 133.67, 136.28, 136.61, 138.66, 151.22, 153.31, 184.11, 184.43, 187.91. M.p. 79.5–81.7 °C. HRMS (ESI) *m*/*z* calcd. For [C_15_H_16_O_3_] [M+H]^+^: 245.1172, found: 245.1163.

The *2,3,8,8-tetramethylnaphthalene-1,4,5(8H)-trione (FRV–5).* Reddish solid (96% yield). ^1^H NMR δ: 1.54 (s, 6H), 2.05 (s, 3H), 2.06 (s, 3H), 6.31 (d, *J* = 10 Hz, 1H), 6.74 (d, = 10 Hz, 1H). ^13^C NMR δ: 12.29, 12.37, 26.34, 38.39, 127.45, 131.04, 141.23, 154.54, 157.48, 183.15, 184.92, 187.47. Mp. 141.1–144.7 °C. HRMS (ESI) *m*/*z* calcd. For C_14_H_14_O_3_ [M+H]^+^: 231.1016, found 231.1018.

The *8,8-diethyl-2,3-dimethylnaphthalene-1,4,5(8H)-trione (FRV–6).* Reddish liquid (87% yield). ^1^H NMR δ: 0.61 (t, *J* = 7.6 Hz, 3H), 1.69 (dq, *J*_1_ = 7.6 Hz *J*_2_ = 13.7 Hz, 2H), 2.0 (d, *J* = 1.0 Hz, 3H), 2.06 (d, *J* = 1.0 Hz, 3H), 2.55 (dq, *J*_1_ = 7.6 Hz *J*_2_ = 13.7 Hz, 2H), 6.52 (d, *J* = 10 HZ, 1H), 6.56 (d, *J* = 10 Hz, 1H). ^13^C NMR δ: 9.45, 12.34, 32.54, 49.11, 131.66, 134.83, 140.92, 141.81, 153.11, 155.56, 183.73, 184.37, 187.40. HRMS (ESI) *m*/*z* calcd. for C_16_H_18_O_3_ [M+H]^+^ 259.1329, found 259.1351.

### 2.3. Cell Lines

The mouse mammary adenocarcinoma TA3/Ha cell line was kindly provided by Dr. Jorge Ferreira, University of Chile, and was grown as described [30]. Human breast cancer MDA-MB-231 and MCF7 cell lines were purchased from the American Type Culture Collection (ATCC, Manassas, VA, USA). RMF621 corresponds to hTERT-immortalized mammary fibroblasts derived from a reduction mammoplasty obtained via a generous gift from Dr. Charlotte Kuperwasser (Tufts University, Boston, MA, USA). TA3/Ha, RMF-621, MCF7, and MDA-MB-231 were grown in Dulbecco’s modified Eagle’s medium (DMEM), containing 25 mM glucose and 4 mM glutamine supplemented with 10% fetal bovine serum (FBS), penicillin (100 IU/mL), and streptomycin (100 μg/mL). The culture media contained no exogenous pyruvate supplementation, and cells were maintained in a humidified atmosphere at 37 °C and 5% CO_2_.

### 2.4. Mitochondrial Respiration

Using TA3/Ha tumor cells, isolated mitochondria (0.5 mg protein/mL) were prepared as described previously [17]. Mitochondrial respiration was measured polarographically at 25 °C with a Clark electrode No. 5331 as described above [17,18]. To determine the effect of quinones on OXPHOS, substrates for Complex I (4.2 mM glutamate + 4.2 mM malate), Complex II (5.0 mM succinate + 0.170 µM rotenone), Complex III (0.30 mM duroquinol), and Complex IV (0.075 mM TMPD + 1.5 mM ascorbate) were used. The state 3u (CCCP-stimulated respiration) and state 4o (proton leak-driven respiration) were induced with 200 nM CCCP and 2 µM oligomycin, respectively, as described [19].

### 2.5. Preparation of Sub-Mitochondrial Particles (SMP) and Evaluation of Complex I Activity

SMP were prepared from isolated mitochondria as described previously [31,32]. Complex I activity was measured according to Estornel et al., 1993 [33]. In brief, mitochondrial protein (0.1 mg/mL) was incubated in assay buffer: 10 mM Tris-HCl, 50 mM KCl, 1 mM EDTA, 2 mM KCN, and 5 µM antimycin A (pH = 7.4). Complex I activity was measured using duroquinone (100 µM), juglone (100 µM), and K_3_[Fe(CN)_6_] (250 µM) as electron acceptors [33] and was expressed as NADH:duroquinone, NADH:juglone, and NADH:FeCN reductase activity, respectively [34,35,36]. For D-state, SMP were incubated at 37 °C for 30 min, 4 °C for 10 min, and 37 °C for 30 min in a dry heating block in the absence of substrates. Then, SMP in assay buffer were incubated for 15 min with DMSO (control) or compounds. The reaction was initiated by adding 75 µM NADH, and the absorbance decrease at 340 nm was monitored at 37 °C. For A-active, the re-activation of thermally deactivated SMP was done by incubation of SMP in an assay buffer with 75 µM NADH for 2.0 min at room temperature [14,15]. Then, the addition of compounds was done.

### 2.6. Determination of Mitochondrial Membrane Potential (∆ψm)

TA3/Ha cells (0.15 × 10^6^ cells/mL) were treated with DMSO (control) or 50 µM FRV–1 and FRV–2 for 2 h. CCCP (200 nM) was used as a positive control. The cells were then washed with PBS and incubated with 50 nM tetramethylrhodamine methyl ester (TMRM, Carlsbad, CA, USA) for 20 min as described [10]. Then, cells were collected, washed, resuspended, and fluorescence was detected using a BD FACSAria III flow cytometer.

### 2.7. Determination of Intracellular ATP, NAD(P)H, and ROS Levels

TA3/Ha cells (1 × 10^5^ cells/mL) seeded into 96-well plates were incubated for 2 h in a culture medium in the absence (control in DMSO) or presence of 50 µM FRV–1 and FRV–2. After exposure, the bioluminescence was measured as described previously using a CellTiter-Glo Luminescent Cell Viability Assay kit (Promega, Madison, WI, USA) [17]. Intracellular NAD(P)H levels were measured through auto-fluorescence using excitation/emission wavelengths of 340 nm/428 nm. In brief, 0.5 × 10^6^ cells/mL were seeded in 96-well plates and incubated in 100 µL PBS for 1 h in the absence (control in DMSO) or presence of compounds (50 µM). Folds change in NAD(P)H content was expressed to the control (DMSO). NAC (5 mM) was used to study the effect of compounds on NAD(P)H and cell viability. The intracellular and mitochondrial ROS levels were determined by dihydroethidium (DHE) and mitoSOX (Invitrogen, Carlsbad, CA, USA) probes, as described previously [7,19]. TA3/Ha cells were incubated for 1 h with DMSO (control), 5 µM rotenone, and 50 µM FRV–1 and FRV–2.

### 2.8. Metabolism Evaluation in Real-Time

The metabolism in real-time analysis of MDA-MB-231 cells was performed in a Seahorse XFe96 Analyzer (Seahorse Agilent, Santa Clara, CA, USA). Cells were seeded (1 × 10^4^ cells/well) on XFe96 V3-PS multi-well plates and kept for 24 h at 37 °C in 5% CO_2_ with a DMEM high glucose culture medium supplemented with 10% FBS. At 24 h, the culture medium was replaced with an assay medium (unbuffered DMEM without red phenol, with 4 mM glutamine and 10 mM glucose, pH = 7.4) 1 h before the assay. Mitochondrial respiration was evaluated by the sequential injection of FRV–1 and analogs at 50 μM, 1 μM oligomycin, 50 nM FCCP, and 1 μM rotenone plus 1 μM antimycin A. The glycolytic function was evaluated by injecting 10 mM glucose, 1 µM oligomycin, and 100 mM 2-deoxyglucose (2-DG), according to Urra et al., 2018 [19]. Oxygen consumption rate (OCR) and extra-cellular acidification rate (ECAR) measurements were made with specific excitation and emission wavelengths of oxygen (532/650 nm) and protons (470/530 nm). Each experiment was performed at least in triplicate.

### 2.9. MTT Assay

The MTT assay was used to evaluate cellular proliferation as described previously [17], seeding 1 × 10^4^ cells/100 µL in 96-well plates and incubating for 24 h. The cells were then treated for 48 h with increasing concentrations of compounds. The OD was measured at 570 nm.

### 2.10. Generation of MDA-MB-231 ρ0 Cells

MDA-MB-231 cells were grown in 6-well plates at 2.5 × 10^5^ cells/well. After 24 h, cells were transfected with 5 µg/mL plasmid pMA3790 (Addgene plasmid #70110) using Lipofectamine^®^ Reagent (Invitrogen, Carlsbad, CA, USA). After 72 h, the GFP-positive cells were selected for FACS and grown in Dulbecco’s modified Eagle’s medium (DMEM), containing 25 mM glucose and 4 mM glutamine supplemented with 10% fetal bovine serum (FBS), penicillin (100 IU/mL), and streptomycin (100 μg/mL), 1 mM sodium pyruvate (GIBCO, Thermo Fisher, Boston, MA, USA) and 50 µg/mL Uridine (Sigma-Aldrich). Confirmation of ρ0 phenotype was performed in Seahorse XFe96. MDA-MB-231 ρ0 cells (2 × 10^4^ cells/well) were seeded on XFe96 V3-PS multi-well plates and kept for 24 h at 37 °C in 5% CO_2_ with DMEM culture medium supplemented with FBS. At 24 h, the mitochondrial function was evaluated by the sequential injection of 1 μM oligomycin, 50 nM FCCP, 1 μM rotenone/1 μM antimycin A, and glycolysis was evaluated with 10 mM glucose, 1 μM oligomycin, and 100 mM 2-DG.

### 2.11. Cell Migration Assay

Cell migration was evaluated in Boyden Chamber assays (Corning, Albany, NY, USA, 6.5 mm diameter, 8 μm pore size) as previously reported [19]. MDA-MB-231 and MDA-MB-231 ρ0 cells were treated with 25 μM FRV–1 for 24 h. Afterward, the cancer cells (50,000 cells/mL) were resuspended in a serum-free medium, plated on top of the chamber insert coated with fibronectin (2 μg/mL), and incubated at 37 °C for 2 h. The inserts were removed, washed, and the bottom side of the inserts was stained with 0.5% crystal violet solution in 20% methanol. Cells from four different frames.

### 2.12. Cell Invasion Assay

MDA-MB-231 cells cultivated in media DMEM (high glucose, without pyruvate) were allowed to migrate for 16 h against 10% FBS using a 6.5 mm Transwell chamber with a pore size of 8 mm (Corning, Corning, Albany, NY, USA) whose membrane was coated with 10 mg of Matrigel on the topside. FRV–1 was added to the upper and lower chamber compartments during the invasion assay. Before these assays, a group of cells was pre-treated for 1 h with mitoTEMPO (1 µM). After invasion time, the membrane was fixed in methanol, and invasive cells were stained on the lower side of the membrane with 0.2% crystal violet [37]. Invasion values are the average of three independent experiments by counting 16 fields from four pictures per chamber.

### 2.13. Zymographic Assay

The secreted gelatinase activity from MDA-MB-231 cells was determined by gelatinolytic zymography, as previously reported [38]. Briefly, aliquots of 24 h conditioned serum-free medium of cells previously treated with FRV–1 and normalized for the same number of cells were subjected to electrophoresis under nonreducing conditions in 10% SDS–PAGE in gels containing 1 mg/mL of gelatin. Then, SDS was removed by extensive washing in 2.5% Triton X-100 and incubated at 37 °C for 16 h in an activation buffer. Finally, the gel was stained with 5% Coomassie blue dye, and MMP-dependent proteolysis was detected as a clear area in a blue field. These areas were quantified by densitometric analysis using Molecular Imaging Software, Kodak version 4.0 (Rochester, NY, USA). Uncropped gels are shown in Supplementary Figure S2.

### 2.14. Electrochemical Experiments

Cyclic voltammetry (CV) was performed using a CH Instrument (CHI 750) electrochemical workstation. All the experiments were carried out with 2.0 mM of each compound at ambient conditions. A stationary glassy carbon electrode (GCE, CH Instrument with an area of 0.0707 cm^2^) was used as a working electrode. The surface of the electrode was polished to a mirror finish with 0.1 µm alumina powder before each experiment, and the surface was cleaned with ethanol in an ultra-sonication bath for 60 s. A platinum spiral wire was used as an auxiliary electrode, and the potentials were measured against a nonaqueous Ag/Ag^+^ reference electrode CH Instrument 112.

### 2.15. Statistics

All statistical analyses were performed using Graph Pad Prism 4.03 (GraphPad Software, San Diego, CA, USA). The data are expressed as mean ± SEM of three independent experiments. Statistical analysis was performed using Student’s *t*-test or one-way ANOVA with Bonferroni’s post-test for pairwise comparisons. The data were considered statistically significant when *p* < 0.05.

## 3. Results

### 3.1. FRV–1, but Not Quinone FRV–2, Is an Inhibitor of the NADH Oxidase Activity of Complex I

**Figure 2:**
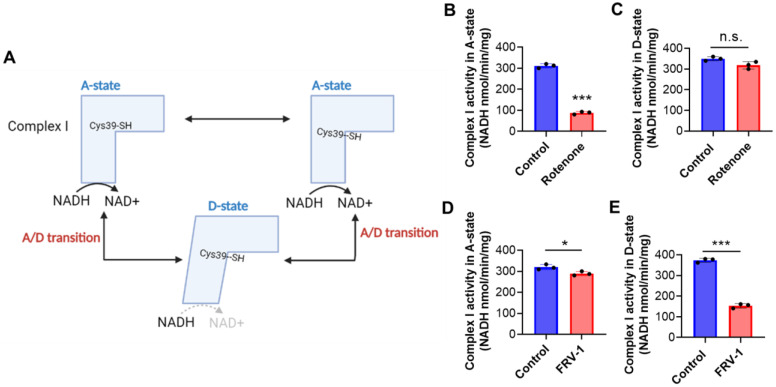
Effect of rotenone and FRV–1 on Active (A-state) and Deactive (D-state) Complex I activity of submitochondrial particles (SMP) from breast cancer cells. (**A**) Current model on A/D transition of Complex I, (**B**,**C**) Effect of rotenone (2.5 µM) and (**D**,**E**) FRV–1 (50 µM) on A- and D-state Complex I activity (NADH: duroquinone oxidoreductase) in SMP from breast cancer TA3/Ha cells. The data shown are the mean ± SD of three independent experiments. * *p* < 0.05, *** *p* < 0.001, vs. Control (DMSO) and n.s.: not significant.

**Figure 3:**
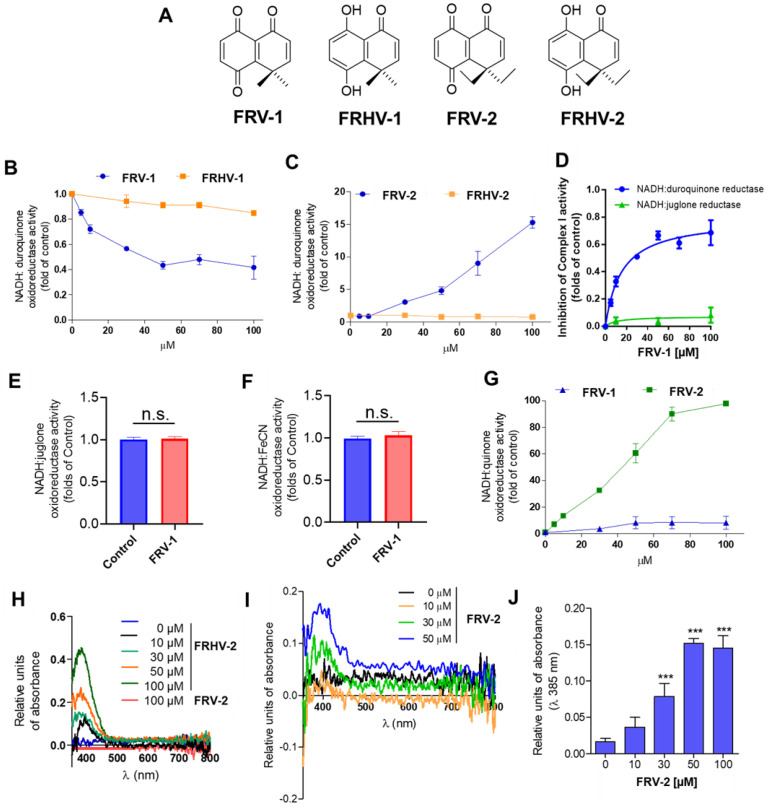
Effects of ortho-carbonyl substituted quinones and hydroquinones on submitochondrial particles from breast cancer cells. (**A**) Chemical structures of ortho-carbonyl substituted quinones and hydroquinones. (**B**,**C**) Effect of quinones and hydroquinones on NADH:duroquinone activity. (**D**–**F**) Inhibition of NADH:duroquinone, NADH:juglone, and NADH:FeCN reductase activity by FRV–1 (100 µM), (**G**) NADH:FRV–1, and NADH:FRV–2 oxidoreductase activity. (**H**) OD spectrum of FRV–2 and FRV–4 (100 µM) in buffer assay, (**I**) OD spectrum of hydroquinone FRV–4 formation (peak: 385 nm) in buffer assay containing SMP, FRV–2, and NADH. (**J**) Quantification of FRHV–2 formation in SMP treated with FRV–2. The data shown are the mean ± SD of three independent experiments. *** *p* < 0.001, vs. Control (DMSO) and n.s.: not significant.

Complex I adopts two catalytically and structurally different states: active (A) and de-active, dormant (D) conformation. In D-state, Cys39 is mainly exposed, and Complex I activity can be re-activated under NADH presence in the A/D transition [10,12] (Figure 2A). In sub-mitochondrial particles (SMP), rotenone (2.5 µM), a known Complex I inhibitor that binds in the ubiquinone-binding pocket, reduced close to 30% the NADH:duroquinone oxidoreductase activity in A-state, lacking the effect on D-state (Figure 2B,C). This result is according to previous reports [14,15]. On the other hand, the quinone FRV–1 (50 µM) slightly reduced the Complex I activity in A-state (Control: 319.6 ± 12.2 vs. FRV–1: 289.0 ± 10.1 NADH nmol min^−1^ mg^−1^, *p* < 0.05) and it exhibited a strong inhibition in D-state (Control: 373.3 ± 9.8 vs. FRV–1: 153.0 ± 10.7 NADH nmol min^−1^ mg^−1^, *p* < 0.001, Figure 2D,E), suggesting that FRV–1 may act on a Complex I conformation-dependent manner. To study this in detail, the effect of quinones FRV–1 and FRV–2 and respective hydroquinones (Figure 3A) was evaluated on NADH:duroquinone reductase activity. Surprisingly, these quinones exhibit different effects on Complex I activity. FRV–1 inhibits Complex I activity, decreasing it to 0.417 ± 0.092-fold of the control (*p* < 0.001) at 100 µM (Figure 3B); in contrast, FRV–2 increases Complex I activity, reaching a maximum effect of 15.26 ± 0.853-fold of the control at 100 µM (Figure 3C). Hydroquinones FRHV-1 and FRHV-2 are inactive on NADH:duroquinone reductase activity (Figure 3B,C). Quinones can interact with greater specificity on different sites in Complex I. These binding sites can be classified as sites before or after the inhibition site of piericidin A [34,35]. To dissect the putative site of inhibition of FRV–1 in Complex I, FeCN (produces the direct re-oxidation of the reduced FMN [39]), juglone, and duroquinone (quinones with binding sites before and after piericidin A, respectively) were used as electron acceptors in enzymatic assays [34,35]. Figure 3D–F shows that inhibition of Complex I activity by FRV–1 is only observed when duroquinone is used as an electron acceptor. This evidence suggests that FRV–1 has a putative inhibition site after the site of action of piericidin A in Complex I in the D-state.

### 3.2. Gem-Diethyl Quinone FRV–2 Is an Electron Acceptor for NADH Oxidase Activity of Complex I

To determine if FRV–2 is a substrate (an electron acceptor) of the NADH oxidation by Complex I, the enzymatic assay was performed in the absence of duroquinone. As Figure 3G shows, FRV–1 increases Complex I activity only 8.25 ± 4.89-fold of the control (at 100 µM), and FRV–2 produces a significant increase of Complex I activity with a maximum close to 100-fold of the control at 100 µM. To confirm that FRV–2 accepts electrons from NADH oxidation catalyzed by Complex I, the occurrence of reduced FRV–2 (hydroquinone FRHV–2) was spectrophotometrically followed. First, in the absence of SMP, the hydroquinone FRHV–2, but does not FRV–2, increases the relative units of absorbance at 385 nm in a concentration-dependent manner (Figure 3H). This allowed us to determine the increase in relative units of absorbance at 385 nm due to the occurrence of reduced FRV–2, a Complex I-dependent event. When SMP plus 75 µM NADH were incubated with FRV–2, an increase in relative units of absorbance compared to the control was observed (Figure 3I,J), confirming that reduced FRV–2 is formed. Taken together, these results suggest that in contrast to FRV–1, FRV–2 is not an inhibitor of Complex I activity in the D-state, but it is an acceptor of the electron transfer involved in NADH oxidation by Complex I.

### 3.3. FRV–1 Inhibits the Complex-I Dependent Respiration in Isolated Tumor Mitochondria

**Figure 4:**
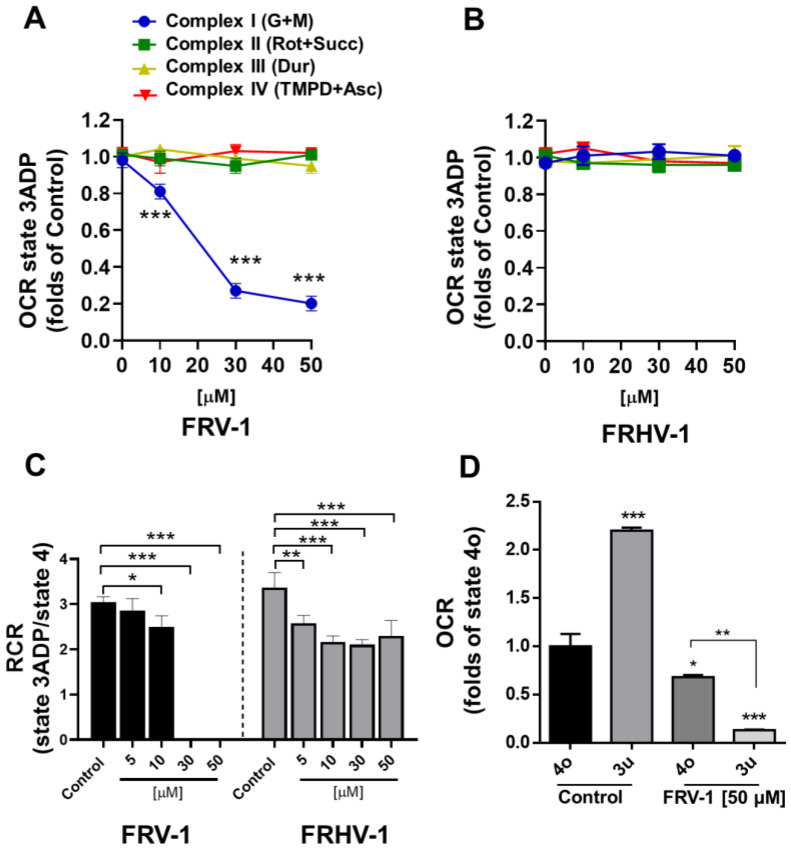
Effect of FRV–1 and FRHV–1 on tumor mitochondrial respiration. (**A**,**B**) Effect of compounds on mitochondrial respiration dependent on each respiratory complex in ADP presence (state 3ADP). (**C**) Effect on compounds on Complex I-dependent respiratory control ratio (RCR). (**D**) Effect of FRV–1 on Complex I-dependent respiration in states 4o and 3u, in the presence of 1 μM oligomycin and 0.2 μM CCCP, respectively. The data shown are the mean ± SD of three independent experiments. * *p* < 0.05, ** *p* < 0.01, *** *p* < 0.001, vs. Control (DMSO).

We selected FRV–1 and FRHV–1 to evaluate the effect of quinone/hydroquinone on respiration in mitochondria isolated from TA3/Ha cancer cells. As Figure 4A,B shows, FRV–1 inhibited the respiration in state 3ADP stimulated by glutamate plus malate (at 50 µM FRV–1: 0.201 ± 0.04 vs. Control: 0.980 ± 0.04, *p* < 0.001), lacking effects on respiration stimulated with substrates for Complex II (succinate), Complex III (duroquinol), and Complex IV (TMPD plus ascorbate) and for FRHV–1 no effects on mitochondrial respiration were observed. In mitochondrial respiration stimulated with glutamate plus malate, FRV–1 induced a reduction of Respiratory Control (RC) until total inhibition at 30 µM (RC = 0 ± 0, vs. Control: 3.04 ± 013, *p* < 0.001). FRHV–1 the RC reduced by 30% without producing OXPHOS uncoupling or total inhibition of respiration (at 50 µM FRHV–1: 2.30 ± 0.34 vs. Control: 3.37 ± 0.33, *p* < 0.001, Figure 4C). Then, we evaluated the effect of FRV–1 on proton leak (presence of oligomycin, state 4o) and maximal electron flux (presence of FCCP, state 3u) dependent on Complex I-maintained mitochondrial respiration. FRV–1 reduced the proton leak- and FCCP-stimulated mitochondrial respirations (Figure 4D). Collectively, these results suggest that FRV–1 is an inhibitor of Complex I-dependent respiration, lacking the effects on other respiratory complexes.

### 3.4. FRV–1, but Not FRHV–1, Induces Mitochondrial Dysfunction in Intact Cells, Inhibiting the Proliferation in a ROS-Dependent Manner

**Figure 5:**
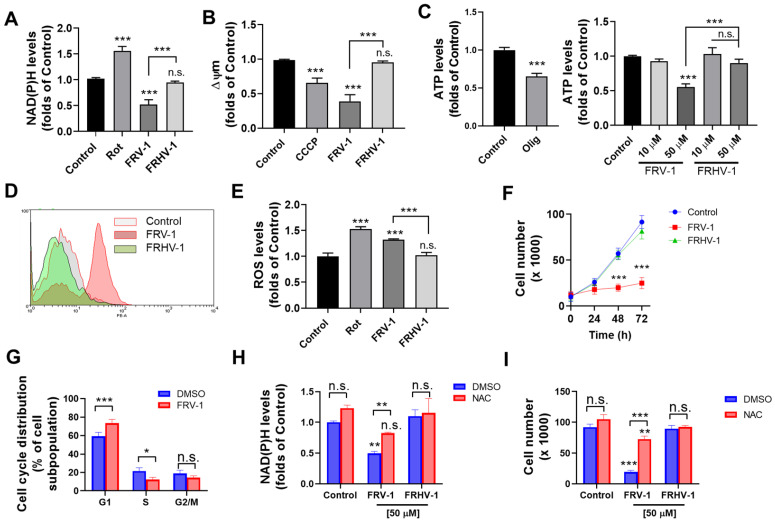
FRV–1, but no FRHV–1, reduces the mitochondrial bioenergetics and inhibits the proliferation of TA3/Ha cells by ROS production. Effects of FRV–1 and FRHV–1 on (**A**) NAD(P)H levels, (**B**) Δψm, (**C**) ATP levels, (**D**) mitochondrial superoxide, and (**E**) intracellular ROS levels at 2 h of treatment. (**F**) Effect of FRV–1 and FRHV–1 (50 µM) on proliferation and (**G**) cell cycle distribution levels. (**H**,**I**) N-acetylcysteine (NAC) partially prevents the effect of FRV–1 on NAD(P)H and proliferation. Data are expressed as means ± SD of three independent experiments. * *p* < 0.05, ** *p* < 0.01, *** *p* < 0.001 vs. Control (DMSO), and n.s.: not significant, Rot: rotenone, Olig: oligomycin.

We evaluated the effect of FRV–1 and FRHV–1 on mitochondrial bioenergetics of TA3/Ha cells, a cell line highly dependent on glutaminolysis and OXPHOS as described [18]. As Figure 5A–C shows, FRV–1 reduced the Δψm and ATP levels, and FRHV–1 lacked effects. Since the inhibition of Complex I activity can produce electron leak-producing mitochondrial superoxide [9,40], we evaluated the mitochondrial and cytosolic ROS and NAD(P)H levels in TA3/Ha treated with FRV–1 and FRHV–1 for 4 h. Quinone but no hydroquinone produced an increase of mitochondrial (Figure 5D) and cytosolic ROS (Figure 5E) levels. Given these results, we evaluated the effect of these compounds on proliferation and the participation of oxidative stress. As Figure 5F shows, FRV–1, but not FVHR–1, reduced the cell number until 72 h of treatment and arrested the cell cycle in G1-phase (Figure 5G). The ROS scavenger, N-acetylcysteine (NAC), prevented the NAD(P)H decrease (Figure 5H) and antiproliferative effect (Figure 5I). Taken together, these results indicate that FRV–1 produces mitochondrial dysfunction, inhibiting the proliferation in a ROS-dependent manner.

### 3.5. FRV–1 Inhibits Mitochondrial Respiration, Inducing a Metabolic Shift in MDA-MB-231 Cells

**Figure 6:**
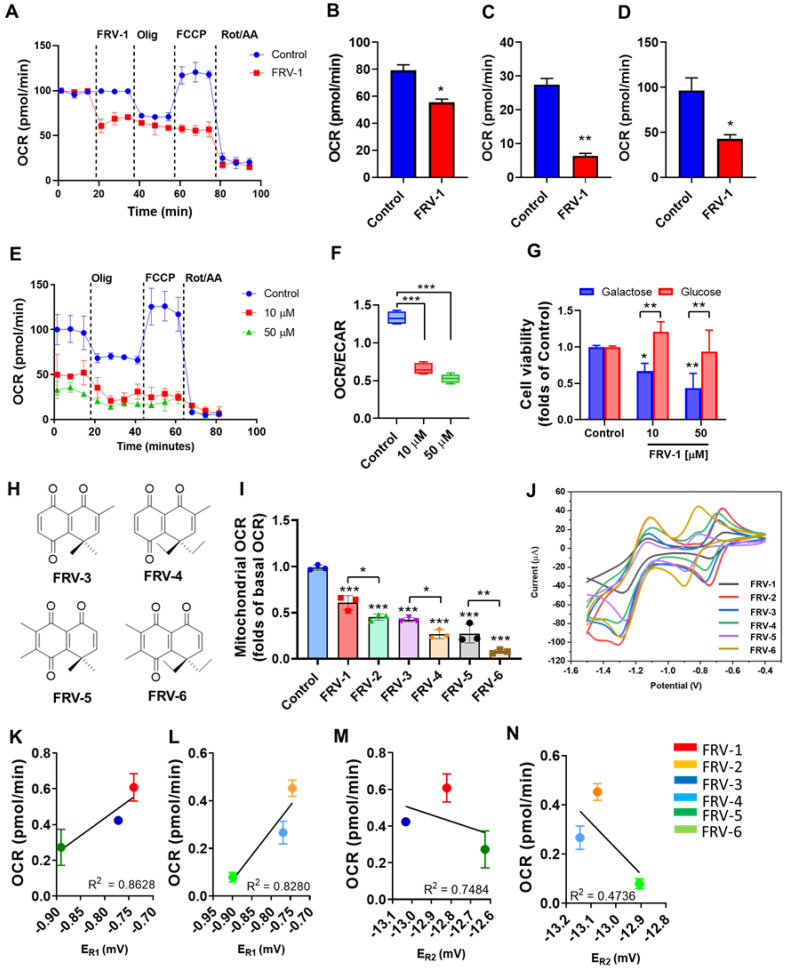
FRV–1 inhibits mitochondrial respiration, triggering a metabolic shift toward glycolysis in MDA-MB-231 cells. (**A**) Effect of FRV–1 on mitochondrial respiration when injected, reducing the (**B**) basal OCR, (**C**) ATP-driven OCR, and (**D**) maximal OCR. (**E**) Effect of FRV–1 on the profile of respiration and (**F**) OCR/ECAR ratio at 4 h of treatment. (**G**) Differential effect of FRV–1 on oxidative (galactose) and glycolytic (glucose) subpopulations of MDA-MB-231 cells. (**H**) Chemical structures of methylated analogs of FRV–1 and FRV–2. (**I**) Effect of methylated analogs on mitochondrial OCR at 50 µM. (**J**) Cyclic voltagramme for all compounds (2.0 mM) at 100 mV/s, (**K**,**L**) OCR-E_R1_ and (**M**,**N**) OCR-E_R2_ relationships for FRV–1 and FRV–2 and analogs. Data are expressed as means ± SD. * *p* < 0.05, ** *p* < 0.01, *** *p* < 0.001 vs. Control.

To determine the effect of FRV–1 on cell metabolism in real-time, this compound was added in breast cancer cells by XFe96 Seahorse, and changes in mitochondrial respiration were evaluated in MDA-MB-231 cells. As Figure 6A shows, FRV–1 instantly inhibits the oxygen consumption rate, reducing the mitochondrial basal OCR (Figure 6B), ATP-driven respiration (Figure 6C), and maximal respiration (Figure 6D). After 4 h of treatment, FRV–1 completely abolishes mitochondrial respiration (Figure 6E) and increases the OCR/ECAR ratio (Figure 6F). These results suggest that FRV–1 produces a metabolic shift toward glycolysis. To explore whether the effect of FRV–1 on cell viability is dependent on metabolism, we used two metabolically different subpopulations of MDA-MB-231 cells, which were generated by growing in glucose (25 mM) or galactose (10 mM) and glutamine (4 mM) [19]. In these conditions, the viability of glycolytic and highly oxidative MDA-MB-231 subpopulations is selectively sensitive to glycolytic or mitochondrial inhibitors, as we previously reported [7,19]. FRV–1 significantly reduced the viability of the oxidative subpopulation of MDA-MB-231 cells, suggesting that glucose availability promotes a pro-survival response (Figure 6G).

### 3.6. Methylated-FRV–1 Analogs Exhibit Increased Action on Mitochondrial Respiration in MDA-MB-231 Cells

To evaluate the effect of structural modifications to the ortho-carbonyl quinone scaffold on OCR inhibition, analogs with methyl and dimethyl substitutions were synthesized (Figure 6H). As Figure 6I shows, the injection of FRV–1 (gem-dimethyl quinone) and FRV–2 (gem-diethyl quinone) reduce the mitochondrial OCR to 0.61 ± 0.07 and 0.45 ± 0.03 folds of the Control, respectively. Notably, the inhibitory effect on OCR is sequentially increased when methyl and dimethyl substitutions are added to the quinone scaffold. The gem-diethyl quinones are significantly more active than gem-dimethyl quinones, and FRV–6 is the best OCR inhibitor of this series. The action of some quinones on respiratory complexes can be determined by the redox potential involved in the one-electron reduction for forming semiquinone radical (E_R1_) and, then, a second one-electron reduction, producing the hydroquinone (E_R2_) [41]. We determined the redox potential of methyl-quinone analogs, as Supplementary Information Table S1 describes and Figure 6J shows. The inhibitory effect on OCR correlates with E_R1_ but not with E_R2_ (Figure 6K–N), suggesting that the formation of the semiquinone radical may be a step essential for the blocking of mitochondrial respiration.

### 3.7. Quinones and Their Methyl-Analogs Exhibit Differential Effects on the Viability of Breast Stromal and Cancer Epithelial Cell Lines

**Figure 7:**
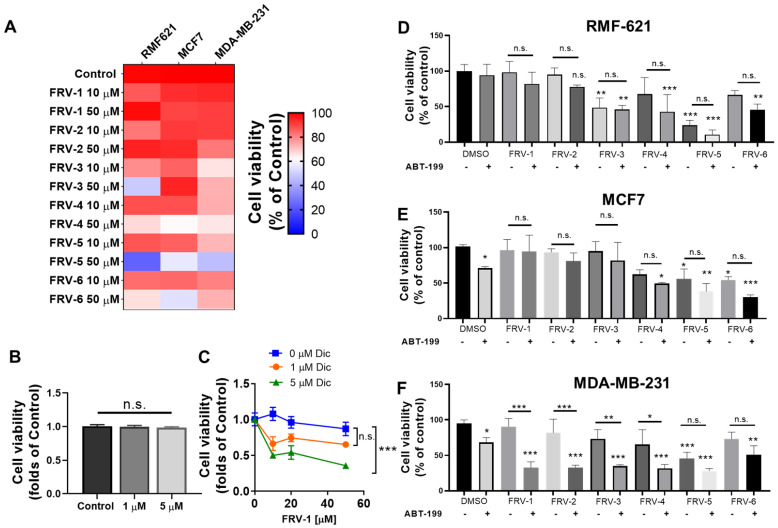
FRV–1 and analogs reduce the viability and produce selective sensitization to BH3 mimetic drug ABT–199 in MDA-MB-231 cells. (**A**) Effect of FRV–1 and analogs on the viability of breast cancer epithelial and stromal cell lines at 48 h of treatment. (**B**) Effect of dicoumarol, an NQO1 inhibitor, on the viability of MCF7 cells at 48 h of exposition. (**C**) Dependence of NOQ1 on the effect of FRV–1 in MCF7. Cells were exposed to NQO1 inhibitor dicumarol (Dic) 1 h before treatment with quinone and viability was evaluated at 48 h of treatment. (**D**–**F**) Effect of combination FRV–1 and analogs (50 µM) with ABT–199 (10 µM). Data are expressed as means ± SD. * *p* < 0.05, ** *p* < 0.01, *** *p* < 0.001 vs. Control, and n.s.: not significant.

We evaluate the effect of FRV–1, FRV–2, and methyl-analogs on the viability of breast fibroblast RMF-621 and two breast cancer cell lines: MCF7 (naphthoquinone oxidoreductase 1, NQO1 positive) and MDA-MB-231 (NQO1 negative) [42]. The IC50 values are shown in Supplementary Table S2. As Figure 7A shows, FRV–3 and FRV–5 reduced the viability of RMF-621 cells at 50 µM, and FRV–4 and FRV–6 preferentially reduced the viability of MCF7 cells at 50 µM. FRV–1 and FRV–2 poorly reduced the viability of stromal and cancer epithelial cells. These results suggest that methyl substitutions in the naphthoquinone ring produce cytotoxic analogs; however, gem-dimethyl, methyl-quinone analogs were compounds with more toxic effects compared with gem-diethyl, methyl-substituted analogs. On the other hand, NQO1 is a quinone-metabolizing enzyme highly expressed in MCF7 [43], which can promote resistance to quinone-induced cell death [44]. Using dicoumarol, a competitive NQO1 inhibitor [45], we evaluated the role of NQO1 in the effect of quinone FRV–1 on the viability of MCF7 cells. Dicumarol or FRV–1 did not affect the viability (Figure 7B,C); however, both dicumarol plus FRV–1 combinations were cytotoxic compared to the FRV–1 condition (Figure 7C), suggesting that this quinone is an NQO1 substrate, being inactive in MCF7 cells.

### 3.8. FRV–1 and FRV–2 Induce Selective Sensitization to BH3 Mimetic Drug, ABT–199 (Venetoclax) in MDA-MB-231 Breast Cancer Cells

The Bcl-2 proteins are less expressed in triple-negative breast cancer cells compared to other BC cells, therefore, being less sensitive to ABT–199 (a BH3 mimetic drug)-induced cell death. Since Complex I activity is a determinant for ABT–199 efficacy in leukemic cells [46], we evaluate the effect of the combinations of methyl-substituted analogs and ABT–199 (10 µM, a non-cytotoxic concentration) on the viability of stromal and cancer epithelial breast cells. Notably, non-significant changes in viability by combinations were observed in RMF-621 and MCF7 cells (Figure 7D,E), which express high NQO1 levels. In contrast, FRV–1, FRV–2, and respective methyl-substituted analogs in combination with ABT–199 reduced the viability in MDA-MB-231 cells (a negative NQO1 cell line, Figure 7F). Notably, these results highlight the selective combinatory effect of FRV–1 and FRV–2 with ABT–199 on MDA-MB-231 cells, lacking the effect on stromal, non-tumoral cells.

### 3.9. FRV–1 Reduces the Migration and Invasion of MDA-MB-231 Breast Cancer Cells in a Functional OXPHOS- and mtROS-Dependent Manner

**Figure 8:**
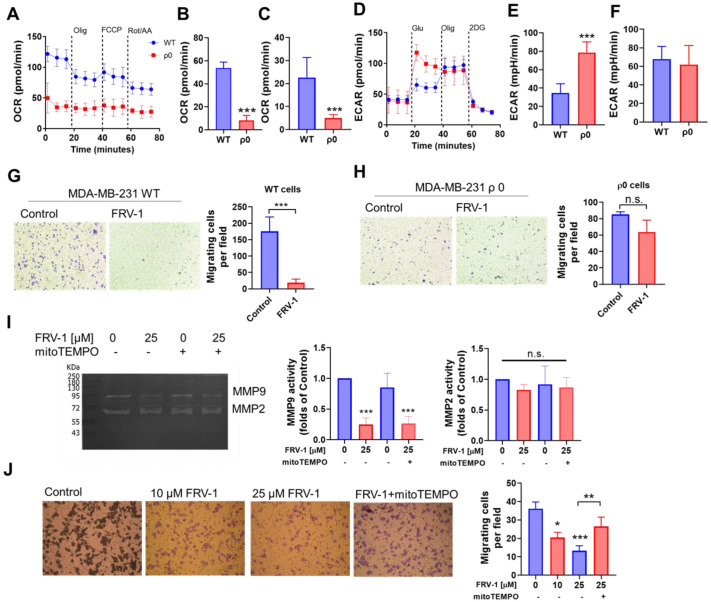
FRV–1 inhibits the migration and invasion in triple-negative breast cancer cells. (**A**) Oxygen consumption rate (OCR) profile of MDA-MB-231 WT (OXPHOS functional) and ρ0 cells (OXPHOS lacking), (**B**) basal, (**C**) maximal OCR (in the presence of FCCP), (**D**) extra-cellular acidification rate (ECAR) profile of MDA-MB-231 WT and ρ0 cells, (**E**) glycolysis, and (**F**) glycolytic capacity. (**G**,**H**) Effect of FRV–1 (25 µM) on the migration of MDA-MB-231 WT and ρ0 cells, (**I**) zymography for MMP activity in MDA-MB-231 WT cells treated with FRV–1 (25 µM), mitoTEMPO (1 µM) and combination and (**J**) invasion of MDA-MB-231 WT cells. Data are expressed as means ± SD. * *p* < 0.05, ** *p* < 0.01, *** *p* < 0.001 vs. Control, and n.s.: not significant.

Mitochondria are essential for supporting the migration and invasion of BC cancer cells [2,19,47], and disruption of mitochondrial respiration elicits migrastatic effects [2,19]. Based on this, we evaluate the effect of FRV–1 on fibronectin-stimulated migration and invasion in MDA-MB-231 cells. To determine whether Complex I is the main target for FRV–1, we generate MDA-MB-231 lacking oxidative phosphorylation (OXPHOS) by mtDNA deletion (MDA-MB-231 ρ0). The ρ0 phenotype was confirmed by metabolism analysis. As Figure 8A shows, ρ0 cells lacked sensitivity to oligomycin, FCCP, and rotenone/antimycin A injections, and MDA-MB-231 Wild type (WT) exhibited a functional OXPHOS (Figure 8B,C). Moreover, ρ0 cells exhibited increased glycolysis compared with WT cells (Figure 8D,E) but without changes in the glycolytic capacity (Figure 8F). MDA-MB-231 ρ0 cells exhibited a minor migratory ability than WT cells, which is consistent with the essential role of mitochondrial electron flux during the metastatic cascade [4]. Using MDA-MB-231 WT and ρ0 cells, we observed that FRV–1 strongly reduced the migration in WT cells (Figure 8G) and lacked the effects on ρ0 cells (Figure 8H), suggesting that the migrastatic effect of FRV–1 is mediated by disruption of the mitochondrial electron flux in OXPHOS competent-breast cancer cells. On the other hand, FRV–1 reduced the proteolytic activity in the 92-kDa-band, corresponding to matrix metalloproteinase 9 (MMP9), without changes in MMP2 activity (72 kDa-band), and consistently with this, it decreased the invasion in MDA-MB-231 WT cells (Figure 8I,J). The addition of mitochondrial antioxidant mitoTEMPO (1 µM) only reversed the anti-invasion effect of FRV–1, suggesting that different (mtROS-dependent and mtROS-independent) mechanisms are triggered by FRV–1. Collectively, these results suggest that FRV–1, a Complex I inhibitor, exhibits migrastatic effects in a functional OXPHOS and ROS-dependent manner.

## 4. Discussion

The currently known Complex I inhibitors exhibit different mechanisms and binding sites [48]. Although all inhibitors of Complex I reduce mitochondrial respiration and increase the NADH/NAD ratio, differences in superoxide production may be significant [40,48,49]. Classic Complex I inhibitors and some new small molecules are uncharged, aromatic, and highly hydrophobic small molecules that can putatively interact with the binding site of ubiquinone or other sites, producing competitive and non-competitive inhibitions [50,51,52]. Generally, they have a hydroquinone/quinone motif that interacts with Complex I, and this interaction is highly sensitive to small structural changes in the inhibitors [18,53,54].

Previously, we have described an *ortho*-carbonyl substituted tricyclic hydroquinone that inhibits the Complex I-dependent respiration in isolated mitochondria of tumor cells [17]. Notably, structure–activity relationship (SAR) studies identified chemical determinants for modifying the action on Complex I and obtaining new OXPHOS uncouplers by protonophore mechanism with selective inhibitory effects on proliferation (e.g., compound FRV-4 in this work) and migration (e.g., compound FR58P1a) in BC cells [18,19]. In the *gem*-dimethyl series, the inhibition of Complex I activity is dependent on intramolecular hydrogen bonding [55,56,57], but *gem*-dimethyl replacement by *gem*-diethyl in hydroquinones promotes the loss of inhibitory action on Complex I.

Despite the above, the effect of *ortho*-carbonyl substituted naphthoquinones on Complex I had been less understood [27,58]. In this work, we evaluated the effect of quinone/hydroquinone couples (FRV–1/FRHV–1 and FRV–2/FRHV–2) on the Complex I activity from SMP. Our results suggest that the substitution of the *gem*-dimethyl by *gem*-diethyl changes the action from a Complex I inhibitor (FRV–1), with a putative binding site after the N2 cluster, to a Complex I electron acceptor substrate (FRV–2), which forms the corresponding hydroquinone FRHV–2 (described previously as an OXPHOS uncoupler, [18]). Since both FRV–1 and FRV–2 reduced Δψm and NAD(P)H levels, these effects may be produced by different mechanisms: FRV–1 by Complex I inhibition/mtROS production-dependent NAD(P)H oxidation, and FRV–2 by Complex I-dependent FRHV–2 formation, which increases the NAD(P)H oxidation by OXPHOS uncoupling. On the other hand, it has been suggested that substitutions to the quinone motif modify the reduction potential [59] and, in turn, change the reactivity with the FeS cluster into Complex I [35,60,61]. Although we observed a slight correlation between Er1 and OCR inhibition in MDA-MB-231 cells by methyl-substituted quinone analogs, an additional contributing factor to determine if an *ortho*-carbonyl substituted quinone is an inhibitor/substrate of Complex I may be steric hindrance present in the *gem*-diethyl quinones, because the terminal methyls of the ethyl groups are arranged above and below the plane of the quinone ring [56].

Complex I has two catalytically different conformations, A- and D-active states, essentials for controlling the mitochondrial superoxide production and electron transport flux during mitochondrial respiration [10,13,48]. In this work, we identified FRV–1 as a novel Complex I inhibitor that affects the catalytically inactive D-conformation, in which Cys39 of the ND3 subunit is mostly exposed to oxidant agents [10]. Although various aspects of the chemical requirements of the D-conformation-inhibiting compounds are unknown, we observed that the quinone FRV–2 and hydroquinones FRHV–1 and –2 did not inhibit the NADH:duroquinone oxidoreductase activity, suggesting that steric hindrance to the quinone ring or loss of it limits its action. The A/D transition occurs according to oxygen and NADH availability and, possibly, the main effect of D-state Complex I inhibitors is the blocking or delaying of the re-activation from D-state to A-active as has been recently suggested for metformin, a D-state inhibitor [14,16,49]. Interestingly, FRV–1 inhibits the Complex I activity in D-conformation SMP at 50 µM, a non-toxic concentration for non-tumoral cells and in intact cells, inhibits the respiration from 10 µM at 4 h. Like this difference in concentrations observed for FRV–1, metformin inhibits the Complex I activity (D-state) in SMP at 25 mM and inhibits the mitochondrial respiration in intact cells at 1.9 mM [16]. In Bridges et al., 2014 [16] and our work, similar methodologies were used, and similar differences in potency between SMP and respiration evaluations were observed. Beyond methodology, the implications of these differences on the biological effect of these inhibitors will require future comparative studies with selective Complex I inhibitors with action on A-conformation. 

NAD(P)H:quinone oxidoreductase (NQO1) is a xenobiotic-metabolizing enzyme with cytoprotective roles in normal tissues [62]. Some of these roles involve p53 stabilization [63], high cytosolic NAD+/NADH ratio, an increase of Complex I activity [64], and activation of Sirt1 signaling (an NAD^+^-dependent deacetylase) in response to the inhibition of mitochondrial respiration [65]. In BC cells, NQO1 overexpression can inactive quinone-based chemotherapeutic drugs by reduction to the respective hydroquinone and is associated with poor prognosis [66]. The MCF7 BC cell line expresses high levels of NQO1, which produces cell death resistance to cytotoxic quinones such as thymoquinone [44] but activates other quinones for producing the biologically active hydroquinone such as plumbagin [67], menadione [68], and β-lapachone [68]. In line with this, our results suggest that FRV–1 is a substrate for NQO1, which could form the respective hydroquinone (FRHV–1) that was inactive in SMP and isolated mitochondria. In contrast, FRV–1 selectively increased the sensibility to ABT–199 (venetoclax) and inhibited the migration and invasion in p53-mutant, NOQ1-null MDA-MB-231 cells.

The differential utilization of glycolysis and OXPHOS between proliferating and migratory/invasive cancer cells has highlighted the participation of mitochondria in chemotherapeutics response and metastasis in BC [2]. Some evidence suggests that metastasis involves a metabolic switch toward an enhanced mitochondrial metabolism [3,4], and Complex I inhibitors, such as metformin and other small molecules, can exhibit migrastatic effects in vitro and in vivo conditions [9,69,70,71]. Consistent with this, FRV–1 produced a strong reduction of migration in OXPHOS-competent MDA-MB-231 cells, and it lacked effects on OXPHOS-deficient ρ0 cells, suggesting that the electron transport chain is a main target for FRV–1. Cell invasion is a complex phenomenon whose activity depends on the acquisition of an intracellular motility capacity, modulated mainly by actin cytoskeleton [72] and some peri- and extra-cellular events as the production of proteolytic activity (carried out mainly by metalloproteinases, MMPs) that degrade surrounding extra-cellular matrix (ECM), allowing cells to migrate [73]. Several studies have shown the crucial role of MMPs in numerous invasive cancers, such as breast cancer [74]. Mitochondrial ROS (mtROS) promotes MMPs activity [75], and for this reason, a vast field of research has been opened seeking to relate mitochondrial redox control and malignancy [76]. Our results show that the invasion of MDA-MB-231 cells was inhibited by FRV–1 in a dose-dependent manner and that depends on mtROS production. Using the same experimental conditions, we found a consistent inhibition of MMP9 activity under the FRV1 action. Nevertheless, contrary to what we expected, the inhibition of MMP9 proteolytic activity was not reverted by a blocking of mtROS production by mitoTEMPO. Interestingly, these results limit the effects of mtROS to cell proliferation as occurs in TA3/Ha cells. Taking these results into account, we can propose that a multi-step phenomenon as cell invasion (by its complexity) is more susceptible to mtROS modulation than MMPs production. Although the cell signaling triggered by FRV–1 via Complex I blocking remains no reported, the ROS production is shown as an essential event in the antiproliferative and anti-invasive effects of FRV–1. Further studies are required to characterize the anti-cancer mechanism of FRV–1.

## 5. Conclusions

Since Complex I contributes to proliferation, metastasis, and chemoresistance, the inhibition of its activity represents an attractive target for anti-cancer strategies. In this work, we describe FRV–1, a novel Complex I inhibitor that acts on catalytically inactive, de-active conformation (D-state), possibly blocking the re-activation to A-state. Our results suggest that FRV–1 inhibits the mitochondrial bioenergetics, promoting a metabolic remodeling toward glycolysis and increasing the mtROS production in BC cells. These effects induce cell cycle arrest in G1-phase, sensitize to ABT–199 (venetoclax) BH3 mimetic drug, and reduce the migration and invasion in NOQ1-null, triple-negative breast cancer cells. 

## Data Availability

The data presented in this study are available on request from the corresponding authors.

## Supplementary Materials

The following supporting information can be downloaded at: https://www.mdpi.com/article/10.3390/antiox12081597/s1. Figure S1: Spectra of the compounds reported; Figure S2: Uncropped gels; Table S1: Values of E_R1_ and E_R2_ for quinones structure of the studied quinones and hydroquinones; Table S2: IC50 values of compounds on RMF621, MCF7, and MDA-MB-231.
