# Supplementary material for: A New Quinone-Based Inhibitor of Mitochondrial Complex I in D-Conformation, Producing Invasion Reduction and Sensitization to Venetoclax in Breast Cancer Cells
Source: Antioxidants (Basel). 2023 Aug 10;12(8):1597. doi: 10.3390/antiox12081597 (PMC10451541; doi:10.3390/antiox12081597)
Supplement: Supplementary file 1 [file antioxidants-12-01597-s001.zip › antioxidants-2446132-supplementary.pdf]

SUPPLEMENTARY INFORMATION

Table S1. Values of E_{R1} and E_{R2} for quinones.

Compound	E_{R1} (V)	E_{R2} (V)
FRV1	-0.7395	-1.2822
FRV2	-0.7451	-1.3074
FRV3	-0.7717	-1.3032
FRV4	-0.7690	-1.3144
FRV5	-0.8906	-1.2627
FRV6	-0.8990	-1.2906

Table S2. Effect of Cpd. 9 and Cpd. 14 on viability of non-malignant (RMF621) and breast cancer (MCF7 and MDA-MB-231) cells. The IC50 values (µM) are the mean of three different experiments ± SD.

**Cpd.**	**RMF621**	**MCF7**	**MDA-MB-231**
**FRV-1**	>100	> 100	> 100
**FRV-2**	95,23 ± 3,01	> 100	> 100
**FRV-3**	50,74 ± 1,25	> 100	> 100
**FRV-4**	67,2 ± 2,03	83,64 ± 3,21	90,91 ± 2,15
**FRV-5**	32,2 ± 2,42	80,27 ± 2,54	62,95 ± 2,17
**FRV-6**	64,7 ± 1,78	64,54 ± 2,37	56,18 ± 1,79

4,4-diethyl-5,8-dihydroxy-2-methylnaphthalen-1(4H)-one (FRHV-4)

1H

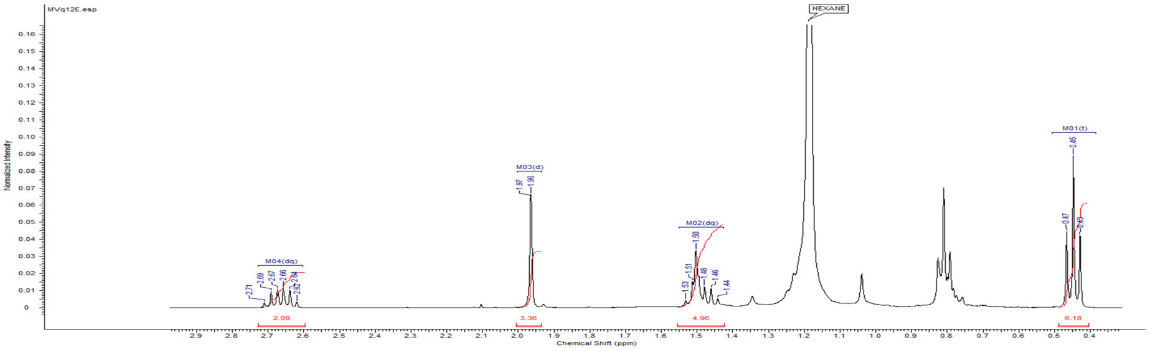

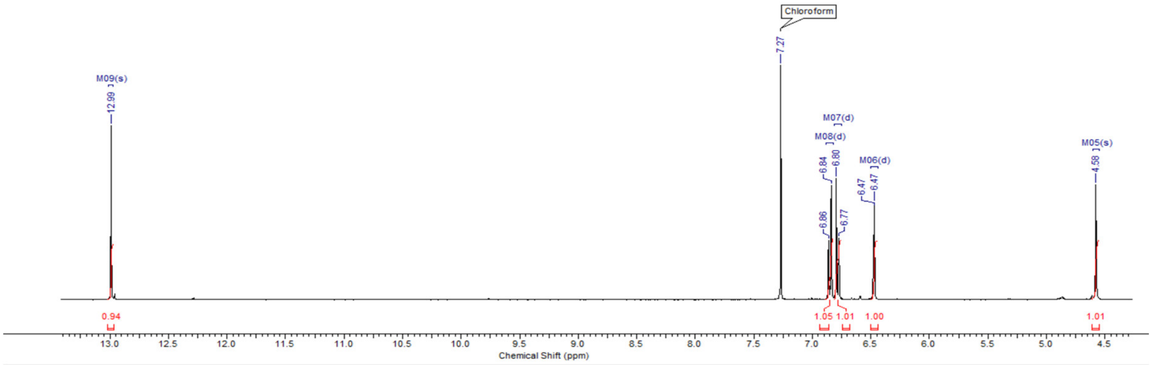

13C

Figure S1. Spectra of the compounds reported.

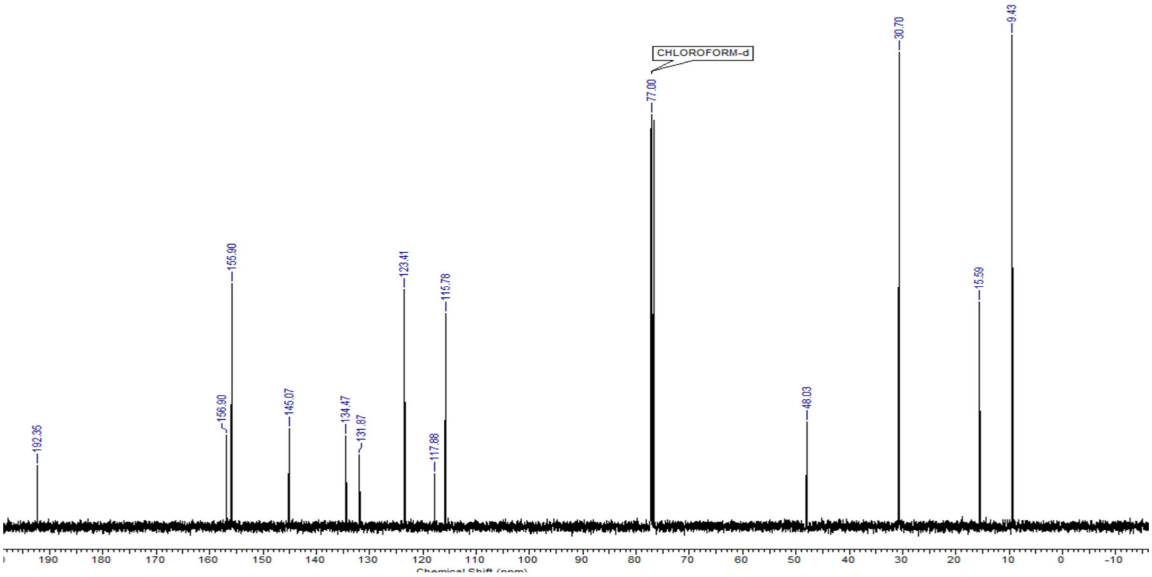

4,4-diethyl-5,8-dihydroxy-6,7-dimethylnaphthalen-1(4H)-one (FRHV-6)

1H

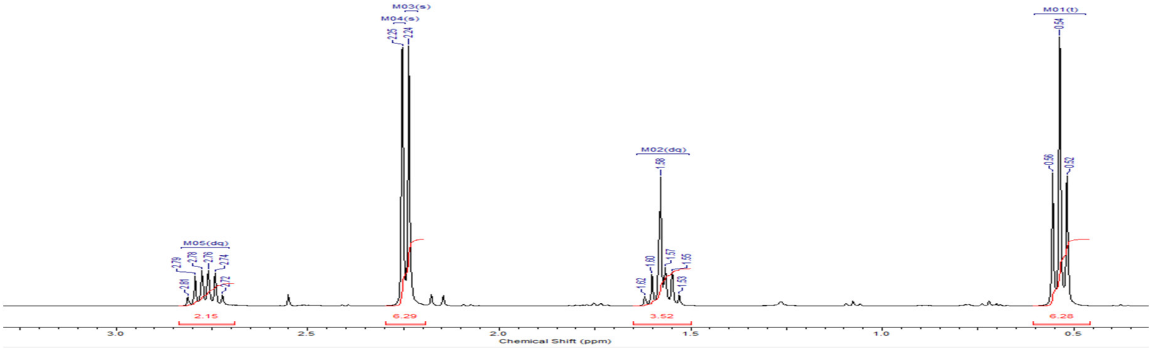

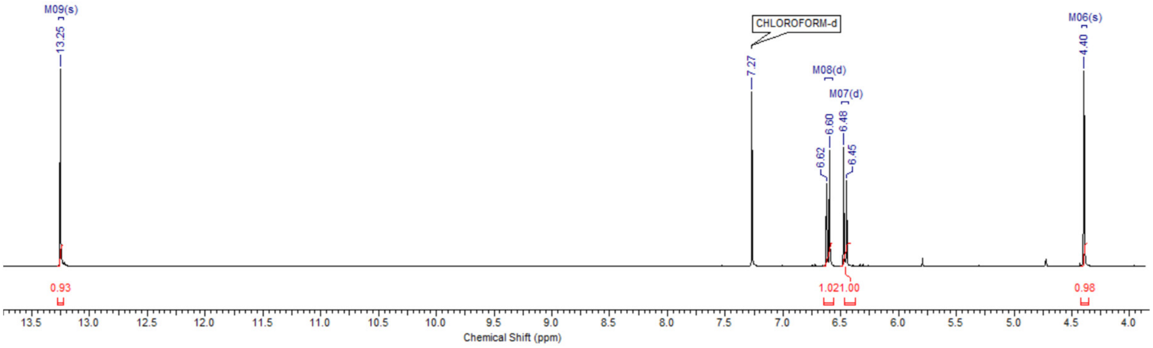

13C

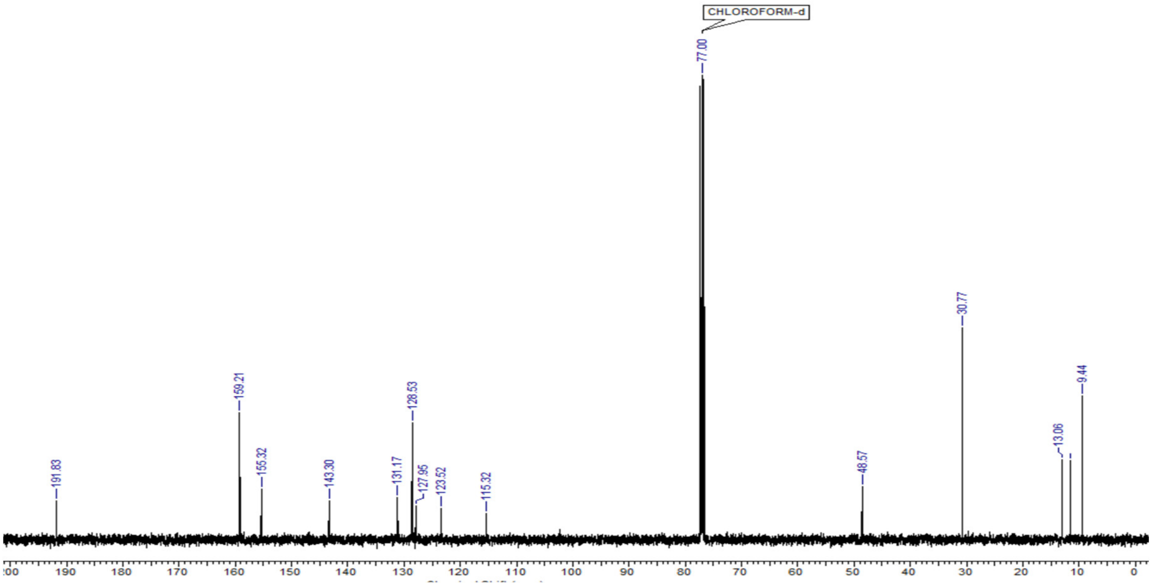

8,8-diethyl-6-methylnaphthalene-1,4,5(8H)-trione (FRV-4)

1H

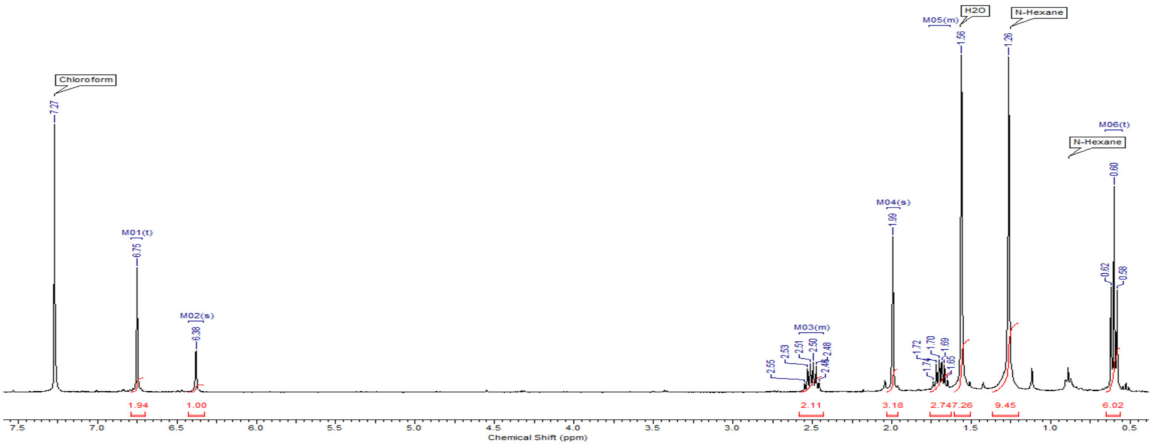

13C

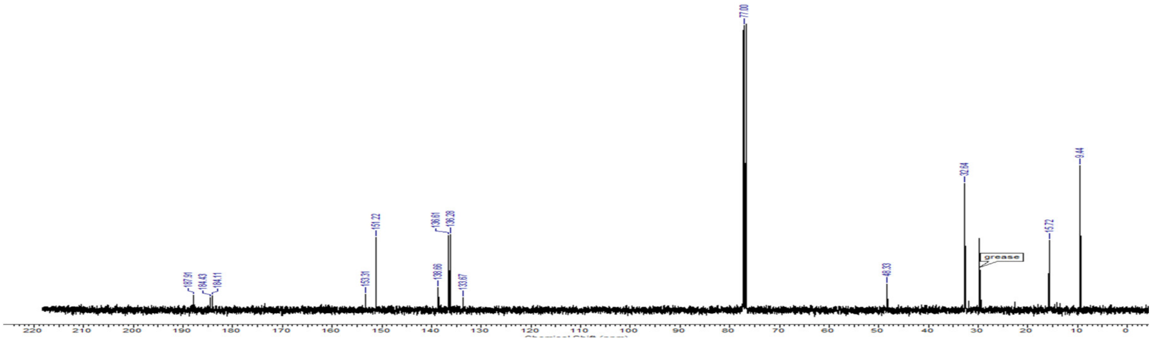
187.91
184.43
184.11
153.31
151.22
138.66
136.61
136.29
133.67
77.00
48.33
32.64
grease
15.72
9.44
220 210 200 190 180 170 160 150 140 130 120 110 100 90 80 70 60 50 40 30 20 10 0

HRMS

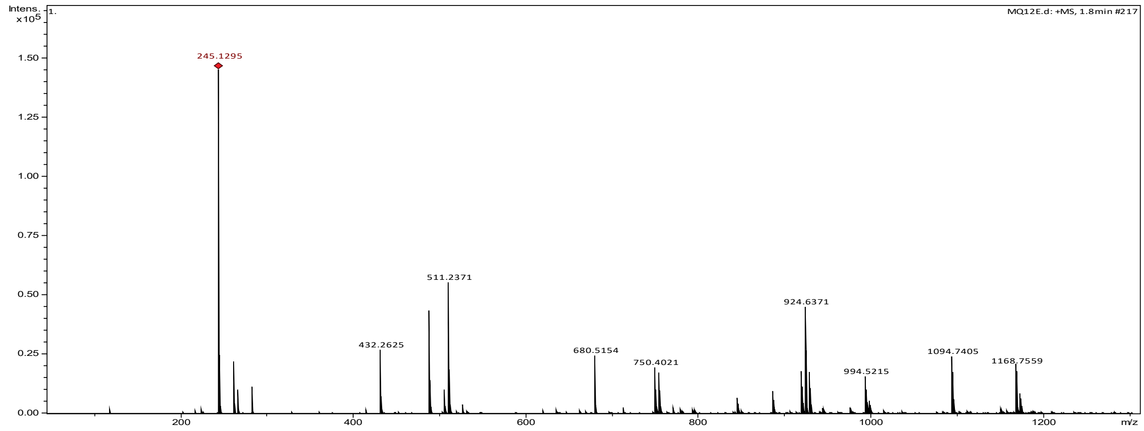
Intens.
x10^5
MQ12E.d: +MS, 1.8min #217
245.1295
511.2371
432.2625
680.5154
750.4021
924.6371
994.5215
1094.7405
1168.7559
1.50
1.25
1.00
0.75
0.50
0.25
0.00
200 400 600 800 1000 1200 m/z

2,3,8,8-tetramethylnaphthalene-1,4,5(8H)-trione (FRV-5).

1H

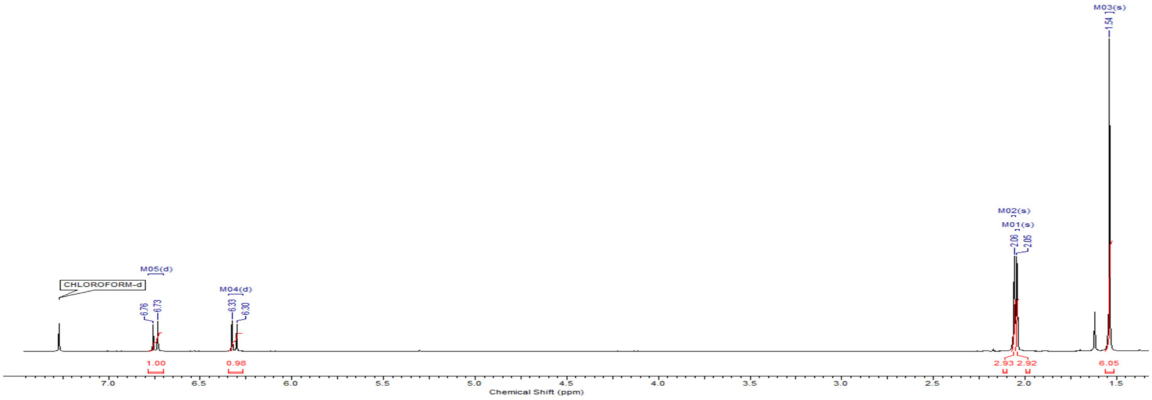

13C

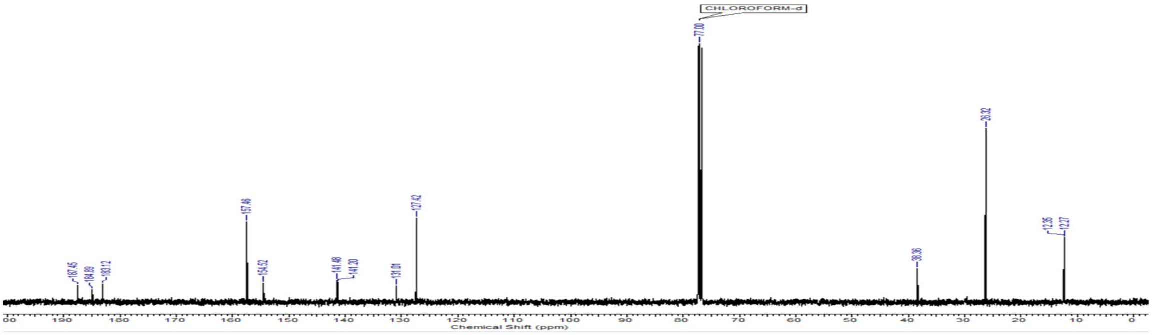

HRMS

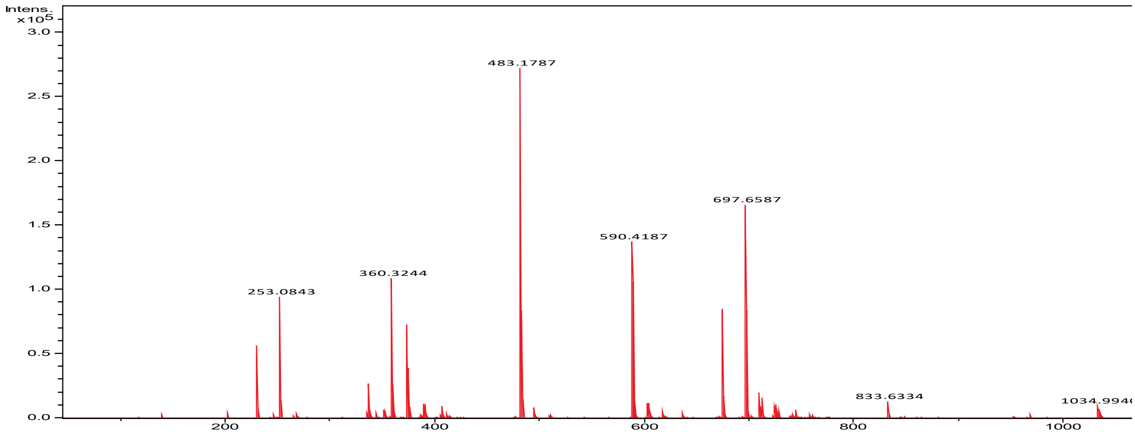

8,8-diethyl-2,3-dimethylnaphthalene-1,4,5(8H)-trione (FRV-6)

1H

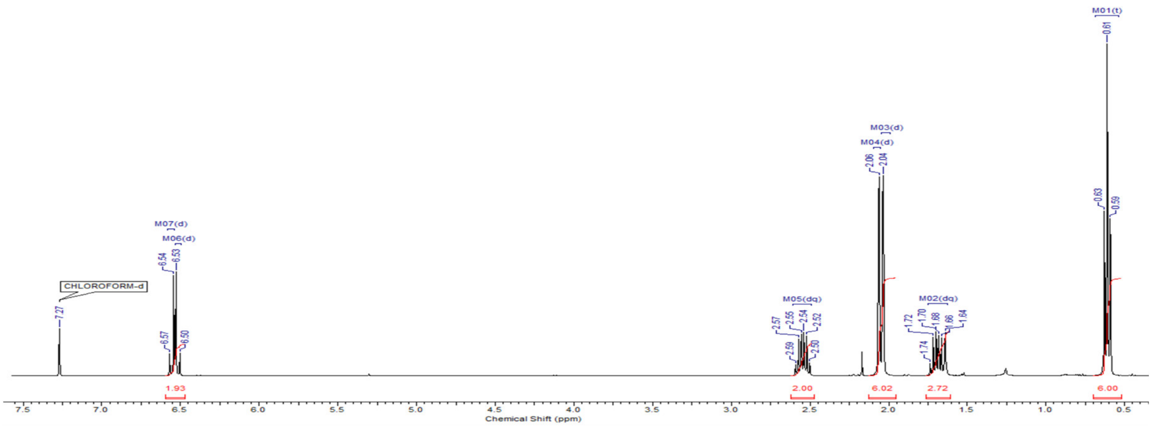

13C

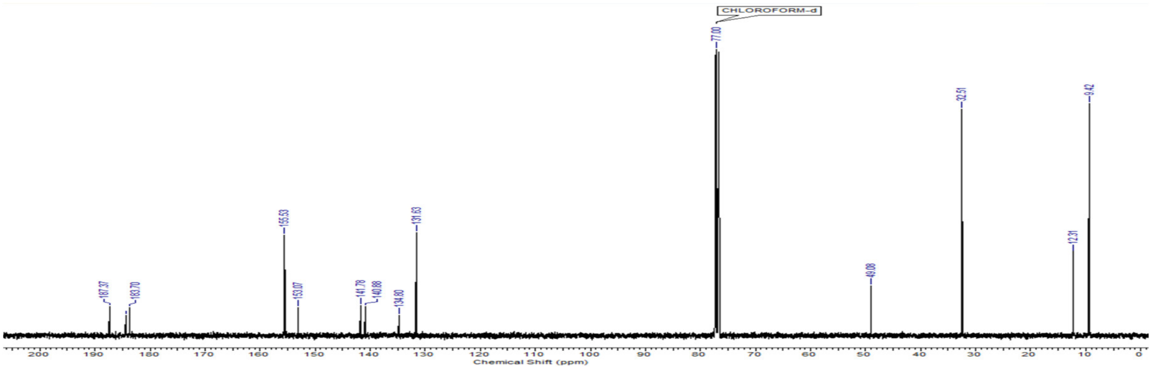

HRMS

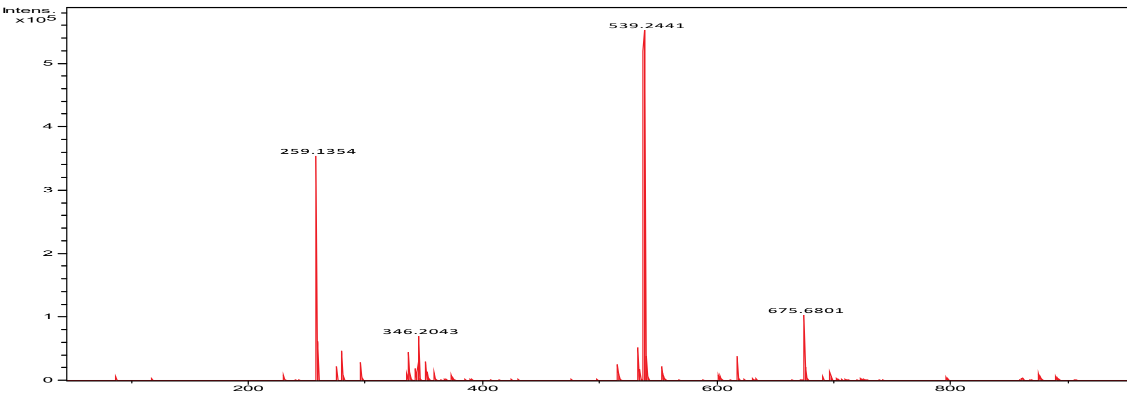

Uncropped gels

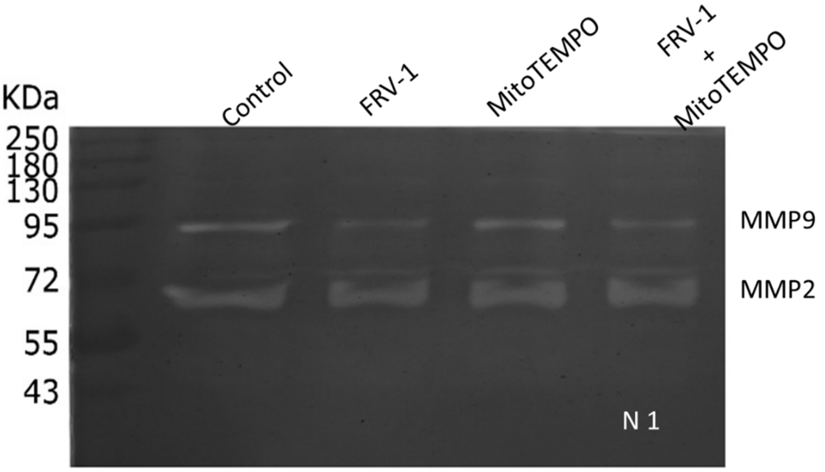

N1 (Used for Figure 8I, representative zymography)

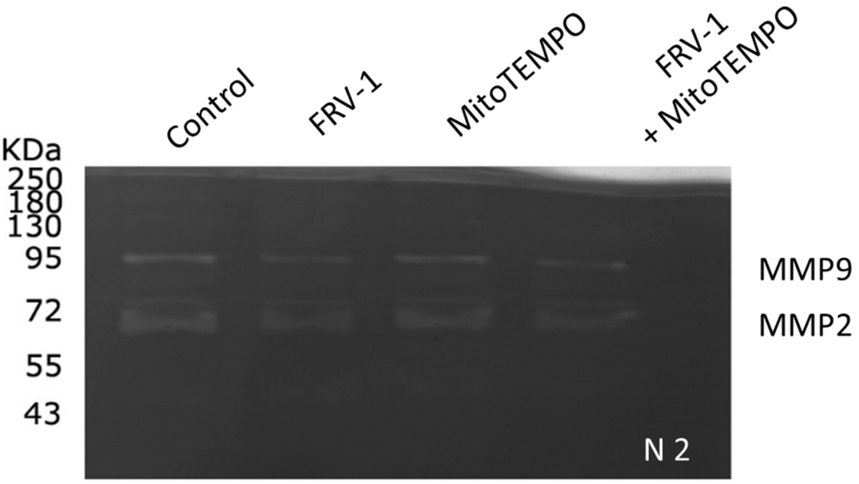

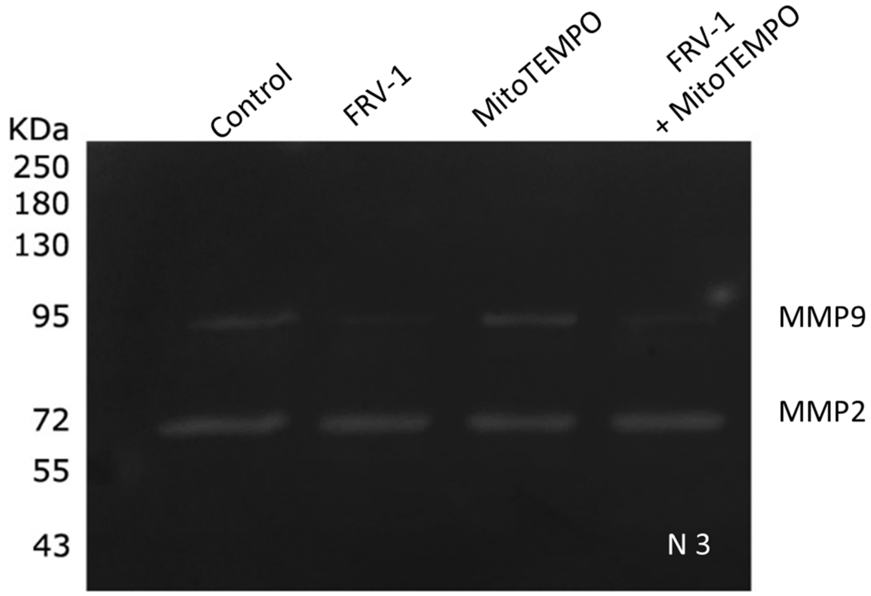

Figure S2. Uncropped gels relative to Figure 8
